# Evaluation of the blood flow in reconstructed gastric tube and its relation to anastomosis leakage

**DOI:** 10.1007/s11748-024-02038-6

**Published:** 2024-05-15

**Authors:** Seigi Lee, Hiroshi Sato, Yutaka Miyawaki, Kazuhiko Hisaoka, Kazuya Takabatake, Tetsuro Toriumi, Gen Ebara, Hirofumi Sugita, Shinichi Sakuramoto

**Affiliations:** Department of Gastrointestinal Surgery, Saitama Medical University International Medical Center, 1397-1, Yamane, Hidaka, Saitama 350-1298 Japan

**Keywords:** Esophagectomy, Anastomotic leakage, Blood circulation, Regional tissue oxygen saturation

## Abstract

**Objectives:**

Anastomotic leakage in esophageal cancer surgery may be reduced by evaluating the blood flow to the reconstructed organ, but quantitative evaluation of arterial and venous blood flow is difficult. This study aimed to quantitatively assess blood flow using a new technique, as well as determine the relationship between the blood flow in the gastric tube and anastomotic leakage using near-infrared spectroscopy.

**Methods:**

This single-center, observational study included 50 patients aged 51–82 years who underwent radical esophagectomy with gastric tube reconstruction for esophageal cancer between June 2022 and January 2023. Regional tissue oxygen saturation was measured at the antrum (point X), the anastomotic point (point Z), and the midpoint between points X and Z (point Y) before and after gastric tube formation. These three points of oxygen saturation were investigated in relation to anastomotic leakage.

**Results:**

When comparing the presence of leakage to its absence, regional tissue oxygen saturation at points X and Z after gastric tube formation was significantly lower (X: *p* = 0.03, Z: *p* = 0.02), with the decreasing rate significantly higher at point Z (*p* = 0.01). There was no significant difference in the decreasing rate of regional tissue oxygen saturation between points X and Y (X: *p* = 0.052, Y: *p* = 0.83).

**Conclusion:**

Regional tissue oxygen saturation levels may be useful for measuring blood flow and could be a predictor of anastomotic leakage.

## Introduction

Anastomotic leakage is one of three major complications in esophageal cancer surgery [1]; however, only a few studies have evaluated the blood flow in reconstructed organs to reduce its frequency. The blood flow is evaluated by measuring the arterial blood flow in the gastric tube (GT) using visualization by indocyanine green (ICG) fluorescence during surgery [2]. ICG fluorescence can be evaluated qualitatively, but not quantitatively, making it difficult to evaluate venous return.

Another way to evaluate reconstructed GT blood flow is through regional tissue oxygen saturation (rSO2), which is commonly used to evaluate brain blood flow. rSO2 indicates oxidized hemoglobin/total hemoglobin, showing the balance between oxygen supply and consumption [3, 4]. Therefore, rSO2 can be used to quantitatively measure the blood flow which has not been possible before and may show its relationship with anastomotic leakage. Additionally, the measurement of the rSO2 values is simple, easy, and noninvasive, with lower rSO2 values and a higher decreasing rate of rSO2 may be associated with anastomotic leakage.

This study aimed to evaluate the blood flow before and after the formation of a reconstructed GT using regional saturation of oxygen measured by INVOS™ and determine its relationship with anastomotic leakage.

## Patients and methods

### Patients

This single-center, observational study was conducted between June 2022 and January 2023, with 65 patients who underwent subtotal esophagectomy in our hospital being recruited. Of these, 50 patients without esophageal benign disease and reconstructed organs, except for the GT, were included in this study. Preoperative patient characteristics were collected, and rSO2 scores were compared at the three measurement points before and after GT insertion in the presence or absence of anastomotic leakage. Similarly, the rates of decline in rSO2 were compared at the three points before and after GT insertion with or without anastomotic leakage (Clavien–Dindo grade 1 or above).

### Surgical method

Thoracic surgery usually involves subtotal esophagectomy via a thoracoscopic approach. Furthermore, lymph node dissection involves two- or threefield dissection, according to esophageal cancer location, lymph node metastasis, and stage; the reconstruction route is usually the retrosternal GT. Most abdominal surgeries, including abdominal lymph node dissection and GT formation, are performed using hand-assisted laparoscopic surgery.

In the present procedure, the right gastroepiploic artery was preserved, and the greater omentum was dissected approximately 3 cm away from it. Subsequently, the left gastroepiploic artery, vein, and short gastric artery were dissected, and the right gastric artery was dissected after the first branch was identified.

GT formation occurs outside the body. First, the antrum was cut using a curved stapler. Second, the gastric wall was cut using four or five linear staplers. A GT range of approximately 3 cm is called a narrow GT. Esophagogastric anastomosis is defined as a cervical anastomosis using a circular stapler.

### Method for evaluating blood flow

**Fig. 1:**
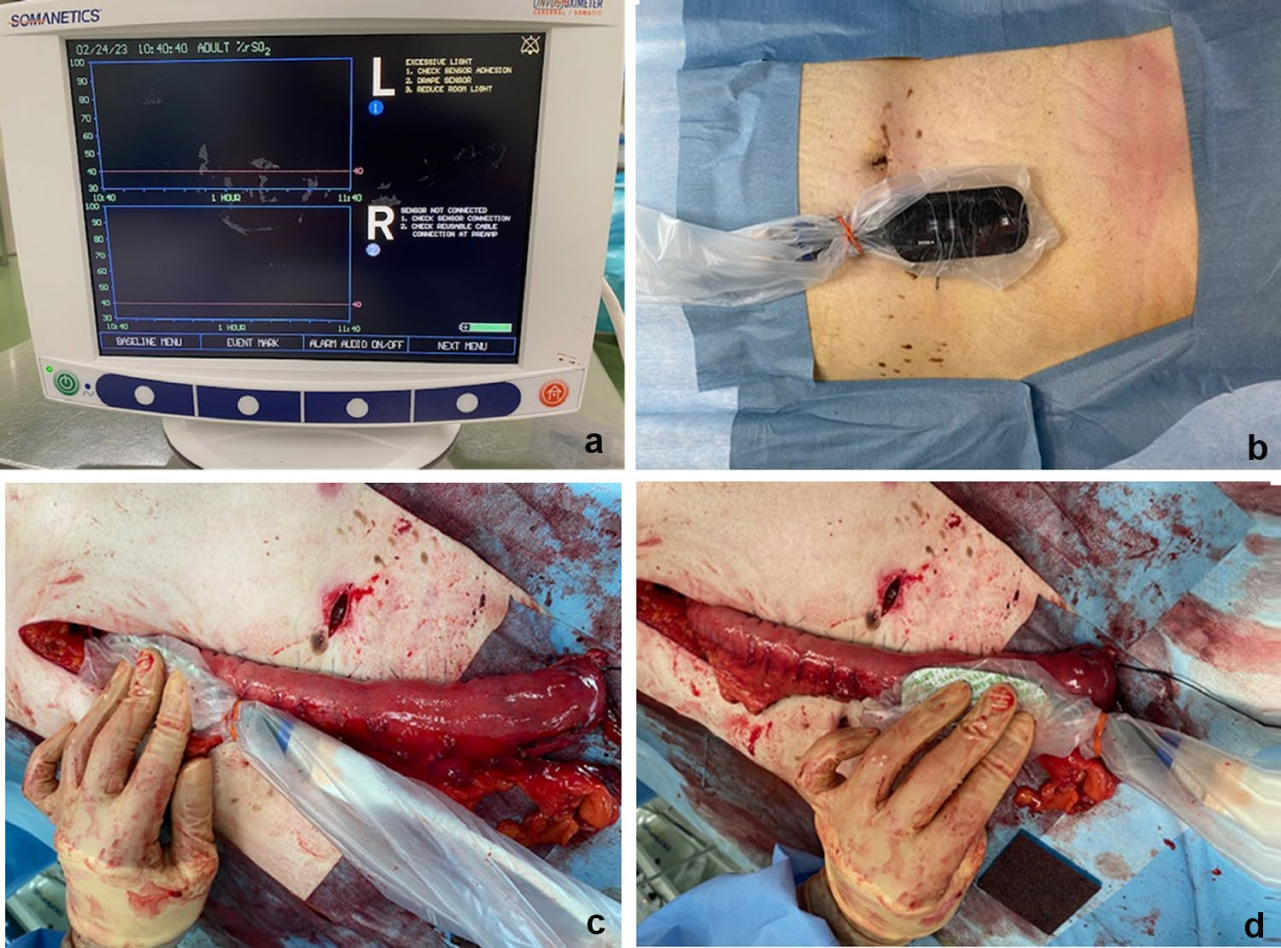
**a** The hemodynamic evaluation device (INVOS™) can measure the regional saturation of oxygen (rSO2). **b** Measuring rSO2 at three points: points X, Y, and Z. **c** rSO2 is measured at the antrum (point X) after GT formation. **d** rSO2 is measured at the anastomotic point (point Z) after GT formation. *rSO2* regional saturation of oxygen

Tissue oxygen saturation of the gastric wall and GT wall was measured with INVOS™ (Medtronic, Minneapolis, MN, USA; Fig. 1a and b), a system that uses near-infrared spectroscopy (NIRS) to measure rSO2. The reconstructed GT can be evaluated by measuring rSO2 at three points: the antrum at point X, the anastomotic point at point Z, and the midpoint of the greater curve between points X and Z at point Y (Fig. 1c, d and e). rSO2 was measured at these points before and after GT formation. The measurements were taken at each point for 20 s at a time, and the stable value was recorded as the measured value. The measurer and recorder were placed separately. Only the recorder judged the measurements, thereby preventing intervention by the measurement.

### Diagnosis of anastomotic leakage

We used esophagography to diagnose anastomotic leakage on postoperative day 7. If there was fever, increased inflammation, or redness of the cervical wound before 7 days postoperatively, the cervical wound was opened, and a diagnosis of anastomotic leakage was made when digestive fluids or pus discharge were present. In addition, if there was leakage of contrast medium outside the anastomosis on esophagography and fever on after postoperative day 7 with abscess formation or air leakage around the anastomosis on CT scan, the diagnosis of anastomotic leakage was made. Anastomotic leakage of Clavien–Dindo grade 1 or above was targeted.

### Statistical analyses

Data were reported as medians or means ± standard deviation, and statistical significance was set at *p* < 0.05. The Mann–Whitney *U* test was used for comparisons between the presence or absence of anastomotic leakage. The association between anastomotic leakage and decreasing rate of rSO2 was examined using the *χ*^2^ test. In addition, multivariate analysis was performed with logistic regression analysis. All statistical analyses were performed using JMP Pro version 16.0 (SAS Institution Inc., Cary, NC, USA).

## Results

Patient characteristics are summarized in Table 1 based on the presence or absence of anastomotic leakage. Anastomotic leakage occurred in the middle thoracic esophagus in all cases (5 out of the 50 patients (10%), wherein four were men and one was women). The mean age of the patients was 68 years (51–82 years). We also identified docetaxel + cisplatin + 5-fluorouracil therapy (leak: *n* = 3, no leak: *n* = 27), cisplatin + 5-fluorouracil therapy (leak: *n* = 1, no leak: *n* = 7), others (leak: *n* = 0, no leak: *n* = 4), or absence (leak: *n* = 1, no leak: *n* = 7) of neoadjuvant chemotherapy and retrosternal (*n* = 4) or posterior mediastinum reconstruction route (*n* = 1).

**Table 1:** Characteristics of case 1

Characteristics of 1	Leakage (*n* = 5)	No leakage (*n* = 45)
Sex		
Male	4	35
Female	1	10
Mean age	68(51–82)	
Tumor location		
Cervical	0	0
Upper thoracic	0	2
Middle thoracic	5	18
Lower thoracic	0	21
Esophagogastric junction	0	4

Characteristics of case 2			
Characteristic 2	Leakage (*n* = 5)	No leakage (*n* = 45)	*p* values
Neoadjuvant chemotherapy			
Docetaxel + cisplatin + 5-fluorouracil	3	27	*p* = 0.63
Cisplatin + 5-fluorouracil	1	7	*p* = 0.69
Others	0	4	*p* = 0.86
None	1	7	*p* = 0.71
Reconstruction route			
Retrosternal	4	43	p = 0.18
Posterior mediastinum	1	2	
Preoperative hemoglobin*	10.9	11.4	*p* = 0.57
Volume of blood loss*	363	261	*p* = 0.41
Albumin*	3.7	3.8	*p* = 0.76
Glycated hemoglobin*	5.9	5.7	*p* = 0.58
Brinkman Index*	370	513	*p* = 0.45

The rSO2 measurements before GT formation at points X, Y, and Z were defined as X1, Y1, and Z1, respectively, while those after GT formation at points X, Y, and Z were defined as X2, Y2, and Z2, respectively.

**Fig. 2:**
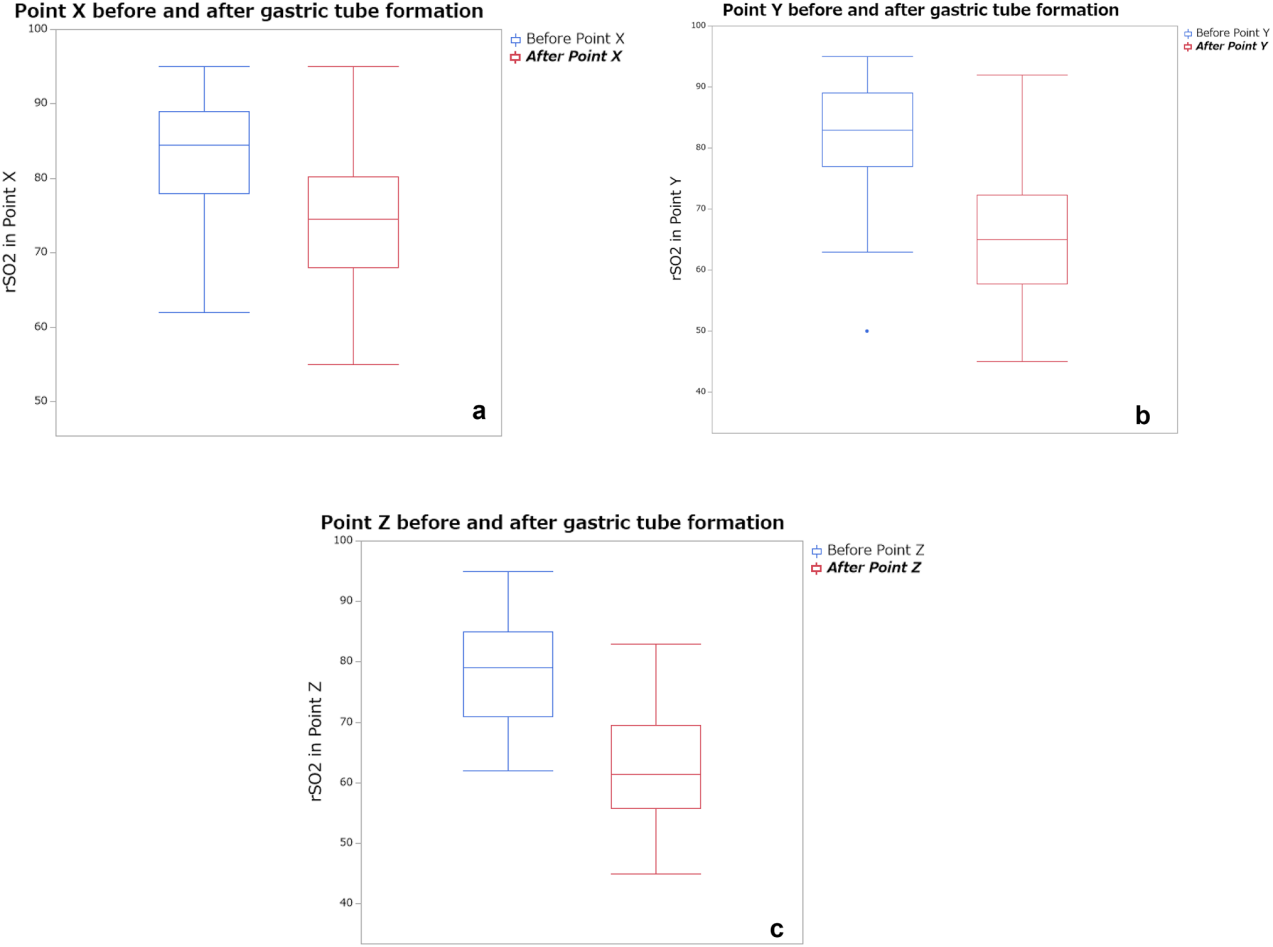
**a** rSO2 values at point X before and after GT formation. **b** rSO2 values at point Y before and after GT formation. **c** rSO2 values at point Z before and after GT formation. *rSO2* regional saturation of oxygen

**Fig. 3:**
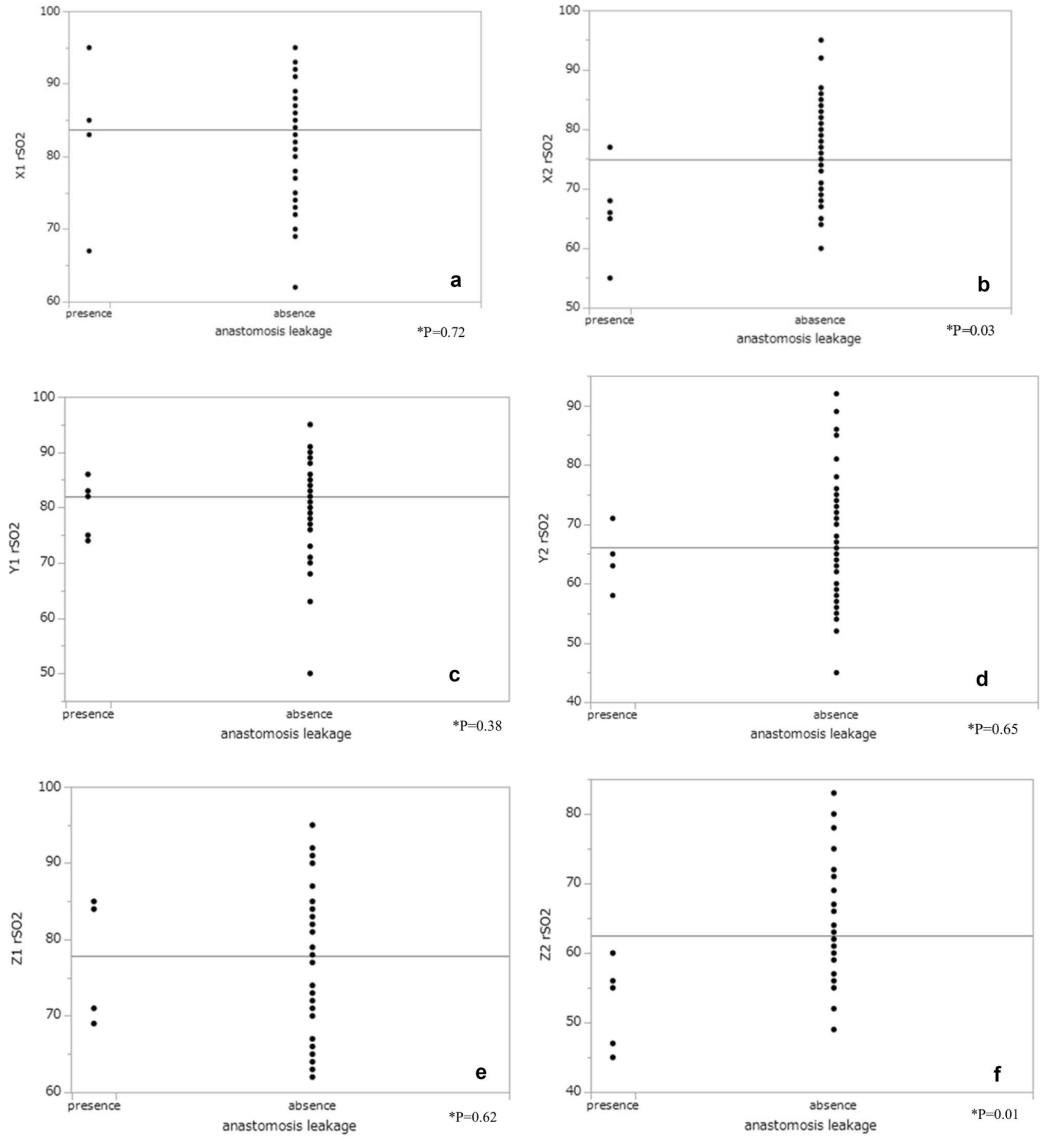
**a** rSO2 measurement at point X before GT formation between the presence and absence of anastomotic leakage. **b** rSO2 measurement at point X after GT formation between the presence and absence of anastomotic leakage. **c** rSO2 measurement at point Y before GT formation between the presence and absence of anastomotic leakage. **d** rSO2 measurement at point Y after GT formation between the presence and absence of anastomotic leakage. **e** rSO2 measurement at point Z before GT formation between the presence and absence of anastomotic leakage. **f** rSO2 measurement at point Z after GT formation between the presence and absence of anastomotic leakage. *rSO2* regional saturation of oxygen

**Fig. 4:**
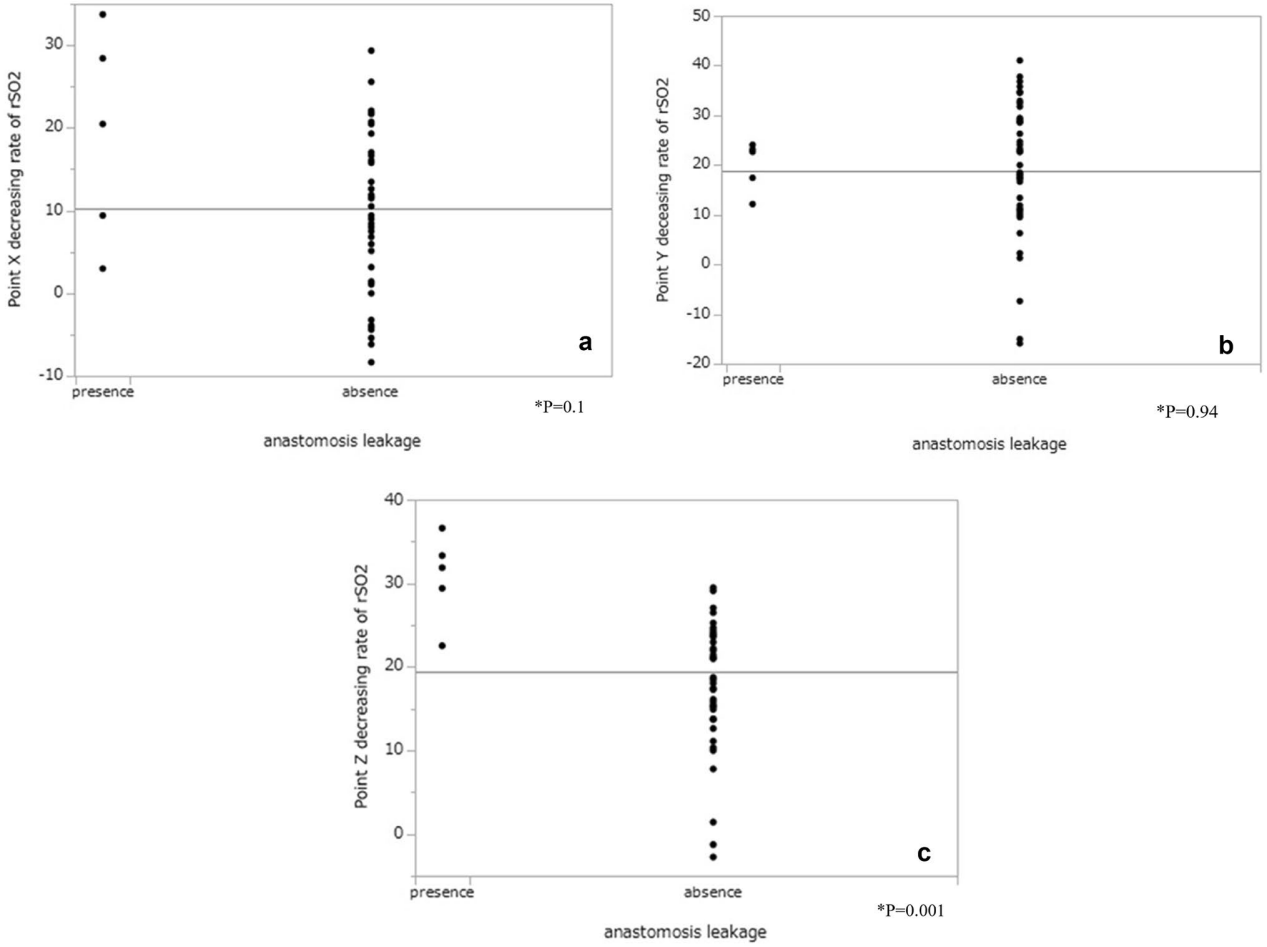
**a** The decreasing rate of rSO2 at point X, **b** point Y, and **c** point Z. *rSO2* regional saturation of oxygen

**Fig. 5:**
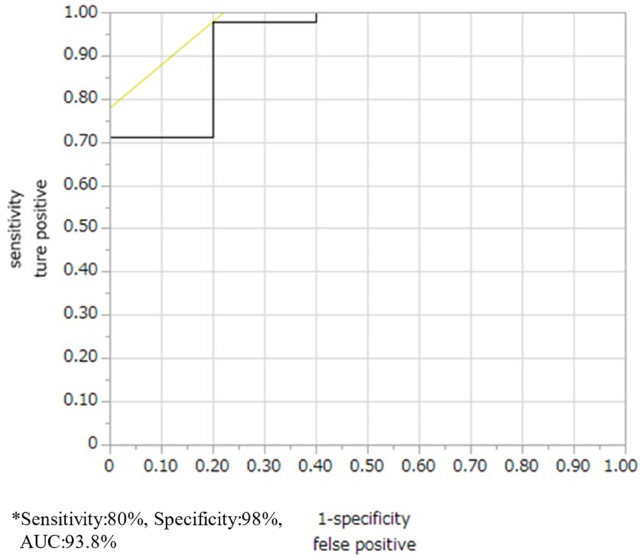
The decreasing rate of rSO2 at point Z RCO curve. The cutoff decreasing rate of the rSO2 was 29.41%. *rSO2* regional saturation of oxygen

The rSO2 measurements at each of the three points are shown in Fig. 2a–c. rSO2 at X2 and Z2 was significantly lower in the presence of leakage than in the absence (X: *p* = 0.03, Z: *p* = 0.02). However, there was no significant difference in rSO2 at X1 (*p* = 0.76), Y1 (*p* = 0.63), Z1 (*p* = 0.66), and Y2 (*p* = 0.62) between the presence and absence of leakage (Fig. 3). At point Z, the decreasing rate of rSO2 was significantly higher in the presence than in the absence of leakage (*p* = 0.01), but at points X (*p* = 0.052) and Y (*p* = 0.83), there was no significant difference in the decreasing rate of rSO2 between the presence and absence of leakage (Fig. 4). Multivariate analysis using logistic regression analysis showed a significant difference only in the decreasing rate of rSO2 in point Z (Table 2). The cutoff values for the decreasing rate of rSO2 and the incidence of anastomosis leakage at point Z would be obtained using ROC curves, the cutoff decreasing rate of the rSO2 was 29.41% (sensitivity: 80%, specificity: 98%, AUC: 0.94; Fig. 5).

**Table 2:** Multivariate analysis using logistic regression analysis for anastomosis leakage

	Univariate				Multivariate			
	Odds ratio	Lower 95%	Upper 95%	*p* value	Odds ratio	Lower 95%	Upper 95%	*p* value
Point X before GT formation	0.98	− 0.13	0.1	0.76				
Point X after GT formation	0.84	− 0.38	− 0.03	0.03*	0.9	− 0.31	0.7	0.23
Point Y before GT formation	0.98	− 0.11	0.08	0.63				
Point Y after GT formation	0.98	− 0.13	0.07	0.62				
Point Z before GT formation	0.98	− 0.13	0.08	0.66				
Point Z after GT formation	0.78	− 0.5	− 0.07	0.02*	0.79	− 0.78	0.11	0.19
Decreasing rate in point X	1.11	0.008	0.23	0.052				
Decreasing rate in point Y	1.001	-− 0.06	0.09	0.83				
Decreasing rate in point Z	1.6	0.19	0.98	0.01*	1.58	0.14	1.1	0.04

## Discussion

GT reconstruction is the most common type of esophagectomy, which can have serious complications, such as anastomotic leakage, delayed oral intake, and prolonged hospitalization [1]. A narrow GT is usually used because of its association with good elevation and blood flow [5], with the latter being a very important factor in complete anastomosis [6–8]. Thus, various studies have reported the methods of blood flow evaluation for GT formation.

For instance, the Doppler blood flow meter technique [9–11] can evaluate GT blood flow by measuring the arterial blood flow in the gastric wall. The evaluation of blood flow using the ICG fluorescence imaging method has been generally reported in surgery [12–15]; accordingly, it has also been used to evaluate GT blood flow. The ICG method has shown good results in qualitative evaluation, and some studies have shown an impact on the incidence of anastomotic leakage with and without qualitative evaluation [13, 14]. However, both venous and arterial blood flows are involved in anastomotic leakage. Fujioks et al. reported that venous pressure is weaker than arterial pressure, and the gastric tube passes through a narrow pathway, so the distal end of the gastric tube is often congested due to impaired venous return. If congestion continues, irreversible tissue injury can occur due to the reduced tissue tension of oxygen [15]. Thus, evaluation of arterial blood flow and venous return is synonymous with quantitative evaluation by ICG fluorescence imaging, but this has not been shown to be associated with anastomotic leakage [16]. In addition, Yukaya et al. attempted a quantitative evaluation using ICG in the context of fluorescence and extinction flow between two points in gastric tube. The patients were classified into normal type, inflow delayed type, and outflow delayed type. However, there was no significant difference in anastomosis leakage in each group. This may indicate that ICG is good at visually assessing inflows but not accurately assessing outflows. [17] Furthermore, the narrow GT is mostly supplied by the right gastroepiploic artery and vein, with 20% of the tip blood flow in the GT being provided by the capillary vessel of the gastric wall. Therefore, it is necessary to quantitatively evaluate the arterial blood flow and venous return including capillary vessels. To this end, INVOS™ using NIRS can be used to measure rSO2 because it indicates microscopic arterial and venous oxygen saturation, and the state of regional perfusion is represented by capturing changes in the regional oxygen supply balance [18, 19]. Recent reports state that NIRS can measure regional blood flow easily, noninvasively, and repeatedly [20–22]. In the field of plastic surgery, Steele reported the usefulness of NIRS for the evaluation of ischemia in the construction of free flaps [20]. Additionally, the usefulness of NIRS has been reported in flap reconstruction and limb ischemia in vascular surgery. The cutoff score for tissue ischemia was 45% in these reports [21].

Yamaguchi et al. used the new noninvasive blood flow evaluation device “toccare” (Astem Co., Kanagawa, Japan) to evaluate blood flow in the GT [4] and found no significant differences in rSO2 values before and after GT formation; however, the relationship between anastomotic leakage and rSO2 could not be elucidated [4, 22].

In this prospective observational study, we used a similar device to evaluate the venous return and arterial blood flow in the reconstructed GT. The measurement of the rSO2 values was simple, easy, and noninvasive, and we used a narrow GT for good elevation. However, tip blood flow in a narrow GT might be poor. Blood flow and tension are major factors in anastomotic leakage. The rSO2 values at each of the three points were significantly lower before GT formation than after, which indicates that GT formation may reduce blood flow in the gastric wall.

The rSO2 values at the antrum (point X) and anastomotic point (point Z) were significantly lower with anastomotic leakage than without. No significant differences were detected in the rSO2 values at point Y with and without anastomotic leakage. We believe these results were influenced by the lack of reference or normal value of rSO2 for each organ and tissue. In some cases, a high rSO2 might have decreased after GT formation, whereas a low rSO2 might have remained low after GT formation. The decreasing rate of rSO2 was significantly different only at point Z. Given the results of the multivariate analysis, the decreasing rate of rSO2 rather than the actual rSO2 values is more likely to represent congestion or ischemia. This rate at an anastomotic point was significantly higher only before GT formation than after. No study has ever described the decreasing rate of rSO2, and this is a new finding. The decreasing rate of rSO2 may be more important than the rSO2 value as a predictor of anastomotic leakage, and it is recommended that anastomosis be performed at sites with a lower decreasing rate. Anastomosis may reduce anastomosis leakage by performing the sites with the decreasing rate of rSO2 less than 29%. It is also suggested that the addition of superdrainage be considered if the gastric tube is shortened.

This study has limitations. First, the sample size was too small to determine whether the rSO2 values were related to anastomotic leakage. However, the decreasing rate of rSO2 before and after GT formation showed sufficient potential to predict anastomotic leakage, which has not been explored before. Second, the rSO2 values were unstable. It is clear that GT formation decreases GT blood flow. However, we observed cases in which rSO2 decreased at point X, which was thought to be unaffected by reduced blood flow, and rSO2 increased after GT formation. The decrease in rSO2 at point X was thought to be due to the increase in reduced hemoglobin caused by venous stasis due to the decrease in venous return caused by GT formation. However, the increase in rSO2 after GT formation was thought to be due to measurement instability. The different conditions at the time of measurement in each case and the fact that the measuring probe is equipped with a probe cover made the values unstable. To solve these problems, efforts were made to obtain value stability by taking the measurement time. The decrease in rSO2 at Point X after gastroduodenal tube formation is thought to be due to an increase in reduced hemoglobin level due to venous stasis.

In the future, we will analyze more cases to investigate the relationship between rSO2 and anastomotic leakage. The need to increase the credibility of statistical considerations was discussed.

## Conclusion

We evaluated the rSO2 of GT to determine the relationship between GT blood flow and anastomotic leakage. rSO2 may be useful for measuring blood flow and the decreasing rate of rSO2 may be a predictor of anastomotic leakage. Maintaining consistent conditions for rSO2 measurements can be challenging due to the sensitivity of rSO2 to changes in circulating blood flow and oxygen administration. In order to establish more detailed and appropriate rSO2 criteria, an additional and larger study is needed.

## Data Availability

The datasets used or analyzed in the current study are available from the corresponding author upon reasonable request.
